# Lower 24-hour urinary 6-sulfatoxymelatonin and exploratory bile-acid and microbiome associations in adults with dyslipidemia: a prospectively enrolled cross-sectional study

**DOI:** 10.1080/07853890.2026.2743367

**Published:** 2026-10-07

**Authors:** Alexandre Tavartkiladze, Levan Tavartkiladze, Russel J. Reiter, Michel Burnier, Engin Ulukaya, Gaiane Simonia, Dinara Kasradze, Pirdara Nozadze, Pati Revazishvili, Irine Andronikashvili, Maia Maisuradze

**Affiliations:** a Institute for Personalized Medicine, Tbilisi, Georgia; b Department of Oncology, Faculty of Medicine, Tbilisi State Medical University, Tbilisi, Georgia; c Department of Precision Medicine and Molecular Biology, Osaka University, Japan; d Department of Personalized Medicine, Paris-Descartes University, Paris, France; e Department of Cell Systems and Anatomy, UT Health San Antonio, San Antonio, TX, USA; f Service of Nephrology and Hypertension, Lausanne University Hospital (CHUV) and University of Lausanne, Lausanne, Switzerland; g Faculty of Medicine, Istinye University, Istanbul, Turkey; h Department of Internal Medicine, Faculty of Medicine, Tbilisi State Medical University, Tbilisi, Georgia

**Keywords:** Melatonin, dyslipidemia, 6-sulfatoxymelatonin, bile acids, FGF19, gut microbiome, circadian biology, observational study

## Abstract

**Background:**

We tested the primary hypothesis that lower continuous 24-hour urinary 6-sulfatoxymelatonin (6-SMT) is associated with an adverse lipid and inflammatory profile in adults with dyslipidemia. Associations with FGF19, bile-acid composition, and gut microbiome features were secondary and exploratory.

**Methods:**

This prospectively enrolled, single-center cross-sectional study included 577 consecutive adults evaluated at the Institute for Personalized Medicine, Tbilisi, Georgia, during 2020–2025. Each participant completed three non-consecutive 24-hour urine collections. Validity required a measured-to-predicted creatinine-excretion ratio of 0.80–1.20, and the participant-level 6-SMT value was the arithmetic mean of three valid collections. Fasting lipids, hs-CRP, FGF19, bile-acid profiles, and 16S rRNA taxonomic composition were assessed. An internal method-matched but unmatched normolipidemic reference comparator (*n* = 120) was used only for descriptive analyses.

**Results:**

Median 6-SMT was 8.4 μg/24 h (IQR 5.2–12.1). Lower 6-SMT was associated with higher LDL-C (Spearman ρ = −0.38; approximate 95% CI −0.45 to −0.31), triglycerides (ρ = −0.31; −0.38 to −0.23), and hs-CRP (ρ = −0.35; −0.42 to −0.28), and with lower HDL-C (ρ = 0.29; 0.21 to 0.36). FGF19 was above the stated fasting reference range in 489/577 participants (85%; median 312 pg/mL) and correlated inversely with 6-SMT (ρ=-0.42; −0.48 to −0.35). The emphasized microbial taxa correlated positively with 6-SMT. In a post hoc unadjusted statin-stratified sensitivity analysis, the mean difference in 6-SMT for statin users versus non-users was −0.30 μg/24 h (95% CI −0.83 to 0.23; *p* = 0.26). False-discovery-rate sensitivity analysis across the 14 reported reference-comparator microbiome tests retained *q* < 0.05 for each reported comparison; this does not resolve comparator confounding. The exploratory complete-case multivariable model had adjusted R^2^=0.40 but was not interpreted causally.

**Conclusions:**

Lower 24-hour urinary 6-SMT clustered with adverse lipid and inflammatory measures and with exploratory enterohepatic and taxonomic microbiome features. The findings are hypothesis-generating and do not establish a coordinated biological axis, directionality, diagnostic utility, incremental predictive value, or treatment effect.

## KEY MESSAGES

- Three non-consecutive, creatinine-validated 24-hour urine collections were used to derive each participant-level 6-SMT value.
- The primary hypothesis concerned continuous 6-SMT associations with lipid and inflammatory measures; enterohepatic and microbiome analyses were explicitly secondary and exploratory.
- Elevated circulating FGF19 was observed, but production, clearance, direct FXR activity, and downstream signaling efficiency were not measured.
- The unmatched reference comparator, broader cardiometabolic phenotype, circadian confounding, and cross-sectional design preclude causal, diagnostic, or treatment claims.

## Introduction

Melatonin (N-acetyl-5-methoxytryptamine) is traditionally described as the hormone of darkness because of its nocturnal pineal secretion and central role in circadian organization. It also acts as a pleiotropic molecule with antioxidant, mitochondrial, immunomodulatory, vascular, gastrointestinal, and metabolic functions [1–14]. These actions are relevant to cardiometabolic disease, in which oxidative stress, inflammation, altered energy metabolism, and circadian disruption may coexist.

Dyslipidemia is a highly prevalent cardiovascular risk state. A 2025 systematic review and meta-analysis of 206 adult population studies estimated global prevalences of 28.8% for hypertriglyceridemia, 24.1% for hypercholesterolemia, 38.4% for low HDL-C, and 18.9% for high LDL-C [15]. Contemporary management appropriately prioritizes reduction of atherogenic lipoproteins and global cardiovascular risk, yet residual metabolic and inflammatory risk may persist despite lipid-lowering treatment [16,17].

One underexplored question is whether circadian-redox regulation covaries with the enterohepatic and microbial systems that shape lipid homeostasis. Melatonin is a plausible correlate because it is generated in pineal and extrapineal tissues, including the gastrointestinal tract, and has reported relationships with oxidative stress, intestinal physiology, bile-acid signaling, and microbiome composition [5,18–24]. The present study does not assume that these domains form a single coordinated pathway.

Several upstream determinants can influence both melatonin physiology and cardiometabolic measures, including age, adiposity, diabetes, hypertension, medication exposure, diet, sleep timing, nocturnal light, season, and sleep disorders. A cross-sectional analysis must therefore distinguish a focused primary association from exploratory cross-domain correlations and interpret both in the context of residual confounding.

Bile acids and the gut microbiome form an interdependent metabolic network. Primary bile acids synthesized in the liver are transformed by intestinal bacteria into secondary bile acids, and bile acids signal through receptors including the farnesoid X receptor (FXR). Intestinal FXR signaling promotes FGF19 release, which feeds back to the liver to regulate bile-acid synthesis and lipid metabolism [25–30]. Gut bacteria additionally influence bile salt hydrolase activity, 7α-dehydroxylation, short-chain-fatty-acid production, barrier biology, and inflammatory tone [26,30–34]. These established biological relationships provide context, but they do not convert observed correlations into a demonstrated mechanistic sequence.

The primary hypothesis was that lower continuous 24-hour urinary 6-SMT would be associated with higher LDL-C, triglycerides, and hs-CRP and with lower HDL-C in adults with dyslipidemia. Secondary objectives were exploratory: to examine associations with FGF19, bile-acid composition, and selected 16S rRNA taxonomic features, and to compare reported measures descriptively with a method-matched internal reference cohort. The study was not designed to establish a categorical melatonin-deficiency state, a coordinated biological axis, direct FXR dysfunction, temporal directionality, diagnostic performance, or causality.

## Materials and methods

### Study design and reporting framework

This was a prospectively enrolled, single-center observational cross-sectional study of consecutive adults with dyslipidemia evaluated at the Institute for Personalized Medicine, Tbilisi, Georgia, between 2020 and 2025. Participants were enrolled under a defined protocol, and biospecimens and clinical data were collected contemporaneously at enrollment. No longitudinal clinical outcome was a component of the present analysis. Reporting was revised with reference to the STROBE recommendations for cross-sectional observational studies [35].

Study size was determined by the number of consecutive eligible participants enrolled during the defined 2020–2025 accrual period. No prevalence-estimation sample-size calculation was used because the study was not a population survey. The current report includes all 577 participants with complete values for the variables used in the exploratory multivariable model.

### Participants and eligibility

Adults aged 18 years or older were eligible if they could provide written informed consent and had documented dyslipidemia defined by at least one of the following: LDL-*C* > 130 mg/dL (>3.4 mmol/L), total cholesterol >200 mg/dL (>5.2 mmol/L), triglycerides >150 mg/dL (>1.7 mmol/L), HDL-C below sex-specific thresholds (<40 mg/dL [<1.0 mmol/L] in men or <46 mg/dL [<1.2 mmol/L] in women), or current treatment for diagnosed hypercholesterolemia. Eligibility also required willingness to complete repeated 24-hour urine collection and to provide a stool sample.

Lipid-lowering treatment was not an exclusion criterion. Current statin therapy was recorded at enrollment, included in the exploratory multivariable model, and examined in a post hoc unadjusted stratified sensitivity analysis.

Exclusion criteria were selected to reduce confounding of melatonin secretion, bile-acid metabolism, or microbiome composition. They included current melatonin supplementation or use within three months, melatonin-receptor agonists, beta-blocker therapy, current antidepressant therapy, active treatment for sleep disorders, recent shift work or trans-meridian travel, antibiotic use within four weeks, probiotic supplementation within four weeks, known hepatobiliary disease, inflammatory bowel disease, active malignancy or chemotherapy within the previous 12 months, clinically significant renal insufficiency, pregnancy or lactation, and known pineal-gland abnormalities. Eligibility criteria are also summarized in Supplementary Table S1.

### Clinical covariate definitions

BMI was calculated as body weight in kilograms divided by height in meters squared (kg/m^2^). Hypertension was defined as blood pressure ≥140/90 mmHg or an established diagnosis/current antihypertensive therapy. Type 2 diabetes was defined by an established diagnosis/current antidiabetic therapy or fasting plasma glucose ≥126 mg/dL and/or HbA1c ≥6.5%. BMI, sex, and statin exposure were included in the exploratory model. Hypertension and type 2 diabetes were characterized descriptively but were not inserted post hoc into the original cross-domain model because that model had no prespecified causal estimand or directed acyclic graph and the diagnoses overlap with adiposity, lipid, inflammatory, and medication variables. The resulting coefficients are, therefore, not interpreted as causal or fully confounder-adjusted estimates.

Recent shift work, trans-meridian travel, active treatment for sleep disorders, beta-blocker use, antidepressant therapy, and exogenous melatonin exposure were addressed through eligibility criteria. Habitual sleep duration, chronotype, bedtime, nocturnal light exposure, season, actigraphy, and untreated or undiagnosed sleep disorders were not objectively measured.

### Blood sampling and preanalytical handling

After a 12-hour fast, peripheral venous blood was obtained between 08:00 and 09:00 using standardized venipuncture and tourniquet-application/release procedures intended to minimize hemoconcentration. Blood for the fasting biochemical panel was collected into EDTA-Na tubes. Samples were centrifuged at 1600 × g for 15 min; plasma was separated from cells after a mean of approximately 15–20 min and was analytically ready at approximately 25 min after collection. All samples were processed under CLSI-concordant preanalytical procedures, separation was completed within a maximum of approximately 60–70 min, and analyses were performed immediately. No study plasma sample was frozen.

FGF19, fasting lipid parameters, hs-CRP, and bile-acid measurements were derived from the same morning sampling episode. Processing delays were minimized because several biochemical analytes are susceptible to time-dependent changes after collection.

### Twenty-four-hour urinary 6-sulfatoxymelatonin assessment

Urinary 6-SMT was measured in complete 24-hour urine collections by competitive ELISA (IBL International, catalog no. RE54031). No spot urine or first-morning-void sample was included in the analysis. Three non-consecutive 24-hour collections were obtained from each participant at intervals of 1–2 weeks, with inclusion of at least one weekend day whenever feasible. The participant-level 6-SMT value used in all analyses was the arithmetic mean of three valid collections.

Completeness of each 24-hour collection was evaluated using the ratio of measured to age- and sex-predicted creatinine excretion. Predicted creatinine excretion was estimated as 28–0.2 × age mg/kg/day in men and 23.8–0.17 × age mg/kg/day in women. A prespecified measured-to-predicted ratio of 0.80–1.20 was required for validity; collections outside this range were repeated. Interpretation also considered conditions that materially alter creatinine excretion, including cachexia, marked muscle atrophy, limb amputation, unusually high muscle mass, major meat loading or strict vegan/vegetarian dietary patterns, and unstable acute renal dysfunction. None of these conditions was present in the analyzed cohort.

Instruments were calibrated according to the manufacturer’s instructions for each kit lot. Nine reagent lots were used (A344D, A744C, A781D, A789D, A808D, A890D, A901D, A908D, and A911D). All collections from a given participant were assayed within a single run, assay lot was recorded for sensitivity assessment, and the inter-assay coefficient of variation was 8.2%. Assay specifications are also summarized in Supplementary Table S2.

### Internal age-stratified 6-SMT reference distribution

Because no manufacturer-issued age-specific 24-hour excretion intervals were available for this assay, an internal reference distribution was derived from 120 normolipidemic adults aged 21–81 years recruited over one year from the same institutional source population. The reference cohort included physicians, faculty members, and students affiliated with the Institute for Personalized Medicine. Age and sex were recorded; BMI was not available. Aggregate distributions of sex, comorbidities, medication exposure, smoking, alcohol use, and dietary characteristics were not available in the verified comparator summary used for this revision and were not imputed. Collection, preanalytical handling, assay platform, laboratory personnel, and analytic workflow were the same as for the dyslipidemia cohort. Reference participants had normal serum and urinary creatinine.

After logarithmic transformation, provisional age-stratified reference limits were estimated using the CLSI EP28-A3c robust approach with the Horn-Pesce biweight method and bootstrap 90% confidence intervals based on 10,000 resamples [36,37]. For ages 21–44 years (*n* = 72), the robust center was 20.0 μg/24 h, the 5th percentile was 7.6 μg/24 h (90% CI 6.8–8.5), and the 95th percentile was 52.6 μg/24 h (90% CI 47.1–59.2). For ages 45–81 years (*n* = 48), the robust center was 10.5 μg/24 h, the 5th percentile was 3.5 μg/24 h (90% CI 3.1–4.1), and the 95th percentile was 31.5 μg/24 h (90% CI 28.2–35.8). Because each partition contained fewer than 120 individuals and the comparator was unmatched, the intervals were considered provisional and used for descriptive interpretation only. All inferential analyses treated 6-SMT as continuous. Provisional age-stratified reference distributions and available reference-comparator characteristics are detailed in Supplementary Table S3 and Supplementary Table S4, respectively.

### Supportive nocturnal serum melatonin assessment

Serum melatonin was measured at approximately 03:00 by radioimmunoassay (DIAsource) in a supportive subset of 70 participants spanning all age groups. These measurements were not used to define categorical melatonin deficiency and were not included in the primary multivariable model.

### Lipid, inflammatory, FGF19, and bile-acid measurements

Fasting total cholesterol, LDL-C, HDL-C, triglycerides, and hs-CRP were measured using standardized laboratory methods. FGF19 was measured by ELISA as a circulating marker related to intestinal FXR-mediated enterohepatic feedback. Comprehensive bile-acid profiling was performed by LC-MS/MS, quantifying primary, secondary, conjugated, and unconjugated species. The primary-to-secondary and conjugated-to-unconjugated ratios were treated as descriptive compositional measures. They were not interpreted as direct assays of microbial conversion, bile salt hydrolase activity, FXR activity, or downstream signaling efficiency.

Total cholesterol and triglycerides were measured by enzymatic colorimetry, LDL-C and HDL-C by direct homogeneous assays, and hs-CRP by immunoturbidimetry (Roche Diagnostics). The FGF19 ELISA was supplied by R&D Systems. Total bile acids were measured by enzymatic cycling (Diazyme), and the in-house validated LC-MS/MS bile-acid profile quantified 15 species. The assay reference values summarized in Supplementary Table S2 were total cholesterol <5.2 mmol/L, LDL-*C* < 3.0 mmol/L, HDL-*C* > 1.0 mmol/L in men and >1.2 mmol/L in women, triglycerides <1.7 mmol/L, hs-CRP <3.0 mg/L, fasting FGF19 100–200 pg/mL, and fasting total bile acids 0–10 μmol/L.

### Gut microbiome assessment and internal reference comparator

Fecal samples were frozen within two hours. DNA extraction used the QIAamp PowerFecal Pro DNA Kit. The V3-V4 region of the 16S rRNA gene was sequenced on the Illumina MiSeq platform; processing used QIIME2, DADA2 denoising, and SILVA v138 taxonomic assignment. Alpha-diversity metrics included Shannon, Chao1, and Simpson indices, and beta diversity was assessed using Bray-Curtis dissimilarity. Sequencing depth was at least 10,000 reads per sample.

Microbiome and bile-acid comparisons used the same internal normolipidemic reference cohort (*n* = 120). The specimens were processed by the same personnel with the same extraction, sequencing, bioinformatics, and LC-MS/MS workflows. This group was an internal reference comparator, not a concurrently recruited or individually matched control arm; BMI and several potential confounders were unavailable. C. minuta, F. prausnitzii, and A. muciniphila were not preregistered or specified in a formal analysis plan before examination of the microbiome results. They were selected for targeted reporting on literature-based metabolic and barrier relevance [31–34]. Accordingly, these analyses are exploratory and are not presented as an unbiased whole-microbiome discovery analysis. To reduce selective emphasis, all reported species- and phylum-level comparator tests are shown in the Supplementary Material and included in a false-discovery-rate sensitivity analysis.

### Statistical analysis

Continuous variables are reported as mean ± standard deviation or median with interquartile range according to distribution; categorical variables are reported as counts and percentages. The primary outcome was continuous 24-hour urinary 6-SMT. Spearman rank correlation assessed relationships with lipid parameters, hs-CRP, FGF19, and selected microbiome taxa. Approximate 95% confidence intervals for Spearman coefficients were calculated using Fisher z transformation with *n* = 577; these intervals describe statistical precision and not causal or clinical effects. Group comparisons used parametric or nonparametric tests as appropriate.

A post hoc unadjusted statin-stratified sensitivity analysis compared mean 6-SMT in statin users and non-users with Welch’s t test and a 95% confidence interval for the mean difference. The previously reported complete-case linear model included age, LDL-C, FGF19, F. prausnitzii abundance, A. muciniphila abundance, hs-CRP, BMI, sex, and statin use. Because it was a cross-domain association model rather than a prespecified causal model, it is now labeled exploratory and moved to Supplementary Table S10. Complete data were available for all model variables in all 577 participants; no imputation was performed. Predictor rationale and limitations are provided in Supplementary Table S11.

Microbiome and multi-analyte comparisons were secondary and exploratory. As a sensitivity analysis, Benjamini-Hochberg false-discovery-rate adjustment was applied across the 14 reported internal reference-comparator microbiome tests in Supplementary Tables S7-S9. For p values reported only as inequalities, the boundary value (0.0001 or 0.001) was substituted, yielding conservative upper bounds for q values. Two-sided *p* < 0.05 and *q* < 0.05 were used as descriptive thresholds. Analyses were performed in R version 4.2.0 with microbiome-related packages including phyloseq and DESeq2. These analyses do not correct the unmatched-comparator design or unmeasured confounding.

### Ethics

The study was conducted in accordance with the Declaration of Helsinki and approved by the Ethics Committee of Tbilisi State Medical University (approval numbers CN-2021-11 and TX-UT-2021-08). All participants provided written informed consent.

## Results

### Cohort characteristics

The study included 577 consecutive adults with dyslipidemia. Mean age was 54.3 ± 12.7 years; 312 participants (54%) were male. Mean BMI was 28.4 ± 4.2 kg/m^2^, and 78% were overweight or obese. Mean total cholesterol was 6.8 ± 1.4 mmol/L, LDL-C 4.5 ± 1.2 mmol/L, HDL-C 1.2 ± 0.3 mmol/L, and triglycerides 2.3 ± 1.1 mmol/L. Hypertension was present in 398 participants (69%), type 2 diabetes in 156 (27%), and statin therapy in 423 (73%) (Table 1). This metabolic heterogeneity means that the results may reflect a broader cardiometabolic phenotype rather than dyslipidemia in isolation.

**Table 1.** Characteristics of the 577-participant dyslipidemia cohort.

Parameter	Value	Reference / notes
Total participants	577	Consecutive adults with dyslipidemia
Age, years	54.3 ± 12.7	Range 28–79
Male / female, n (%)	312 (54%) / 265 (46%)	
BMI, kg/m²	28.4 ± 4.2	Overweight/obese: 78%
Total cholesterol, mmol/L	6.8 ± 1.4	Reference <5.2
LDL-C, mmol/L	4.5 ± 1.2	Reference <3.0
HDL-C, mmol/L	1.2 ± 0.3	Reference >1.0 men / >1.2 women
Triglycerides, mmol/L	2.3 ± 1.1	Reference <1.7
24-h urinary 6-SMT, μg/24 h	8.4 (5.2–12.1)	Median (IQR), arithmetic mean of 3 valid collections
FGF19, pg/mL	312 (245–398)	Fasting reference 100–200
FGF19 above reference, n (%)	489 (85%)	
C. minuta, relative abundance	0.02% (0.01-0.04%)	Internal reference mean 0.18%
F. prausnitzii, relative abundance	2.1% (1.2-3.4%)	Internal reference mean 8.4%
A. muciniphila, relative abundance	0.3% (0.1-0.8%)	Internal reference mean 2.1%
Hypertension, n (%)	398 (69%)	
Type 2 diabetes, n (%)	156 (27%)	
Statin therapy, n (%)	423 (73%)	

Values are mean ± SD, median (IQR), or n (%) as appropriate.

### Twenty-four-hour urinary 6-SMT distribution

Median participant-level 24-hour urinary 6-SMT, calculated as the arithmetic mean of three valid collections, was 8.4 μg/24 h (IQR 5.2–12.1). Descriptive subgroup means declined with age, from 12.4 ± 3.8 μg/24 h at ages 20–29 years to 5.1 ± 1.8 μg/24 h at age ≥70 years (Supplementary Table S5 and Supplementary Figure S1). The distribution was descriptively lower than the internal age-stratified robust centers, but the unmatched provisional comparator does not support a diagnostic threshold or universal deficiency label. In the post hoc statin sensitivity analysis, mean 6-SMT was 8.1 ± 2.7 μg/24 h in 423 statin users and 8.4 ± 2.9 μg/24 h in 154 non-users; the mean difference was −0.30 μg/24 h (95% CI −0.83 to 0.23; *p* = 0.26).

Supportive 03:00 serum melatonin measurements were available for 70 participants spanning all age groups. These measurements were not used for categorical classification or in the primary regression model.

### Relationship of 6-SMT to lipid and inflammatory markers

Lower 6-SMT correlated with higher LDL-C (ρ = −0.38; approximate 95% CI −0.45 to −0.31; *p* < 0.001), triglycerides (ρ = −0.31; −0.38 to −0.23; *p* < 0.01), and hs-CRP (ρ = −0.35; −0.42 to −0.28; *p* < 0.001), and with lower HDL-C (ρ = 0.29; 0.21 to 0.36; *p* < 0.01). These moderate associations address the primary hypothesis but do not establish directionality, clinical utility, or independence from the broader cardiometabolic phenotype (Table 2).

**Table 2.** Focused associations of 24-hour urinary 6-SMT with primary and exploratory measures.

Domain	Measure	Spearman ρ	Approx. 95% CI	*P* value	Interpretive status
Primary	LDL-C	−0.38	−0.45 to −0.31	<0.001	Moderate inverse association
Primary	HDL-C	0.29	0.21 to 0.36	<0.01	Small-to-moderate positive association
Primary	Triglycerides	−0.31	−0.38 to −0.23	<0.01	Moderate inverse association
Primary	hs-CRP	−0.35	−0.42 to −0.28	<0.001	Moderate inverse association
Exploratory	FGF19	−0.42	−0.48 to −0.35	<0.001	Moderate inverse association
Exploratory	C. minuta	0.51	0.45 to 0.57	<0.001	Moderate positive association
Exploratory	F. prausnitzii	0.48	0.41 to 0.54	<0.001	Moderate positive association
Exploratory	A. muciniphila	0.45	0.38 to 0.51	<0.001	Moderate positive association

Spearman rank coefficients. Approximate 95% confidence intervals were calculated by Fisher z transformation (*n* = 577). They quantify precision of the observed rank correlations, not causal or clinical effects. Lipid and hs-CRP associations address the primary hypothesis; FGF19 and microbiome associations are exploratory.

### FGF19 and bile-acid profile

FGF19 exceeded the stated fasting reference range in 489 of 577 participants (85%), with a median of 312 pg/mL (IQR 245–398). FGF19 correlated negatively with 6-SMT (ρ = −0.42; approximate 95% CI −0.48 to −0.35; *p* < 0.001) and positively with LDL-C, triglycerides, and hs-CRP. The data establish elevated circulating FGF19 and cross-sectional associations; they do not distinguish altered production, altered clearance, compensatory release, receptor activity, or downstream signaling.

Relative to the internal reference comparator, primary and conjugated bile-acid concentrations were higher and secondary bile-acid concentrations lower. The primary-to-secondary ratio was 2.84 ± 0.78 in the dyslipidemia cohort versus 1.18 ± 0.32 in the reference comparator, and the conjugated-to-unconjugated ratio was 4.18 ± 1.12 versus 2.42 ± 0.68, respectively (Supplementary Table S6 and Supplementary Figure S2). These are descriptive compositional differences, not direct functional measurements of bacterial conversion or bile salt hydrolase activity.

### Microbiome reference-comparator findings

Alpha-diversity metrics were lower in the dyslipidemia cohort than in the internal reference comparator: Shannon index 3.24 ± 0.52 versus 3.82 ± 0.41, Chao1 richness 328 ± 91 versus 412 ± 78, and Simpson index 0.88 ± 0.05 versus 0.94 ± 0.03 (Supplementary Table S7 and Supplementary Figure S3). Each reported comparison remained below *q* ≤ 0.0014 in the conservative false-discovery-rate sensitivity analysis, but unmatched-comparator confounding remains.

C. minuta abundance was approximately 9-fold lower, F. prausnitzii 4-fold lower, and A. muciniphila 7-fold lower than in the internal reference comparator (Supplementary Table S8 and Supplementary Figure S4). These taxa correlated positively with 6-SMT: C. minuta ρ = 0.51 (approximate 95% CI 0.45 to 0.57), F. prausnitzii ρ = 0.48 (0.41 to 0.54), and A. muciniphila ρ = 0.45 (0.38 to 0.51), all *p* < 0.001. Across all 14 reported microbiome comparator tests, conservative Benjamini-Hochberg q values remained <0.05 (Supplementary Tables S7-S9). Because the comparator was unmatched, key covariates were unavailable, and 16S rRNA profiling is taxonomic, these differences are exploratory and do not establish microbial function.

### Exploratory multivariable model

In the complete-case exploratory model (*n* = 577), standardized coefficients were largest for LDL-C (β = −0.31), F. prausnitzii (β = 0.26), age (β = −0.24), FGF19 (β = −0.22), and A. muciniphila (β = 0.18); adjusted R^2^ was 0.40. The model was designed to summarize cross-domain covariation, not to estimate causal effects or incremental clinical prediction. It did not include every cardiometabolic diagnosis, and its coefficients should not be described as independent of all plausible confounding. Full coefficients, diagnostics, and the covariate rationale are provided in Supplementary Tables S10 and S11.

### Integrated hypothesis-generating framework

The observed associations can be organized as a hypothesis-generating framework of interrelated circadian, enterohepatic, microbial, lipid, and inflammatory domains (Figures 1 and 2). Figure 2 now displays bidirectional relationships and common upstream determinants, including age, adiposity, diabetes, hypertension, statins, diet, sleep/light exposure, season, and sleep disorders. The framework is not a demonstrated biological axis or mechanistic sequence.

**Figure 1.**
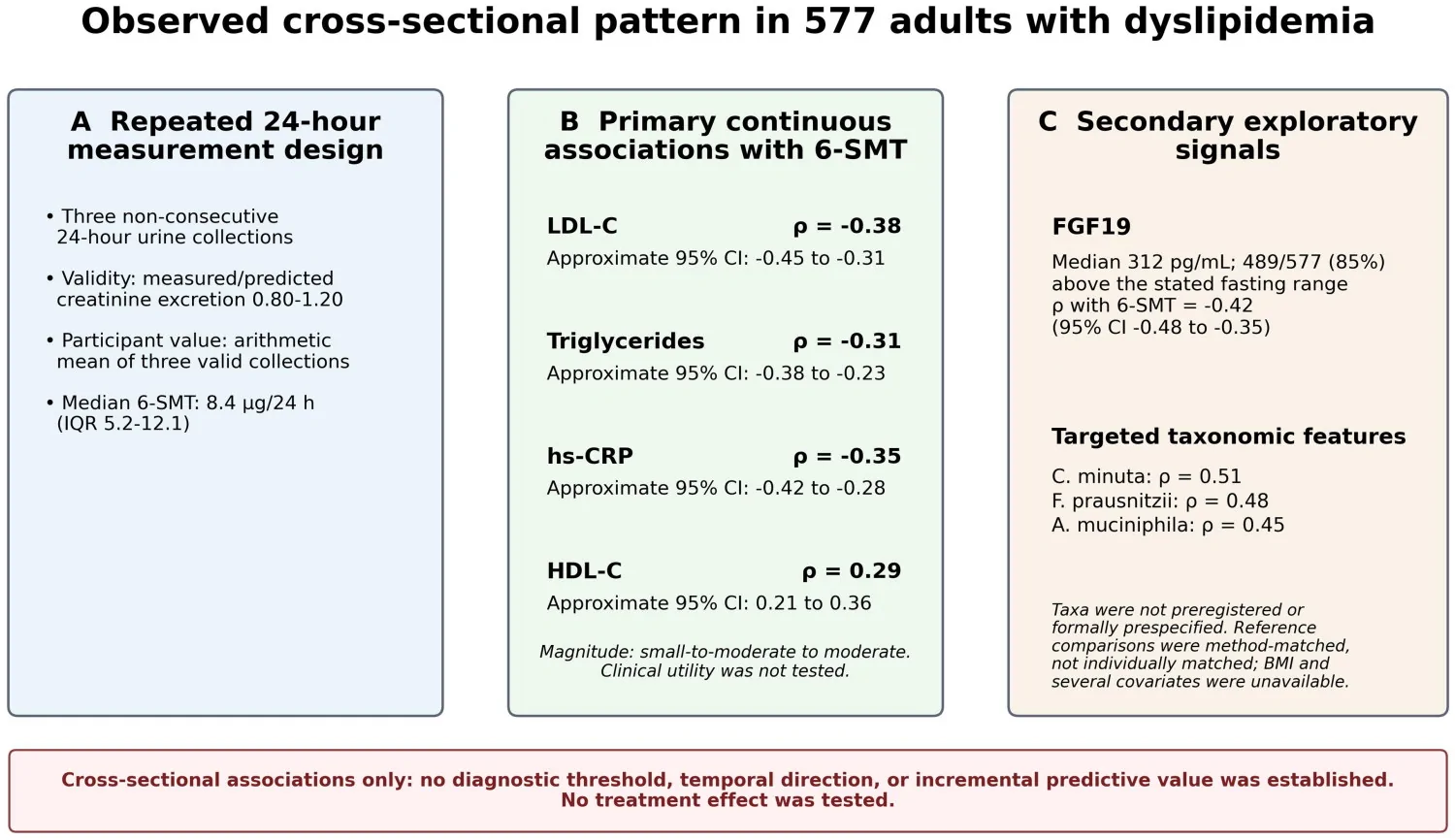
Observed cross-sectional pattern in 577 adults with dyslipidemia. Schematic summary of the repeated 24-hour 6-SMT protocol, focused lipid and inflammatory associations with approximate 95% confidence intervals, and exploratory FGF19 and taxonomic microbiome findings. The figure does not define a diagnostic threshold or imply causality.

**Figure 2.**
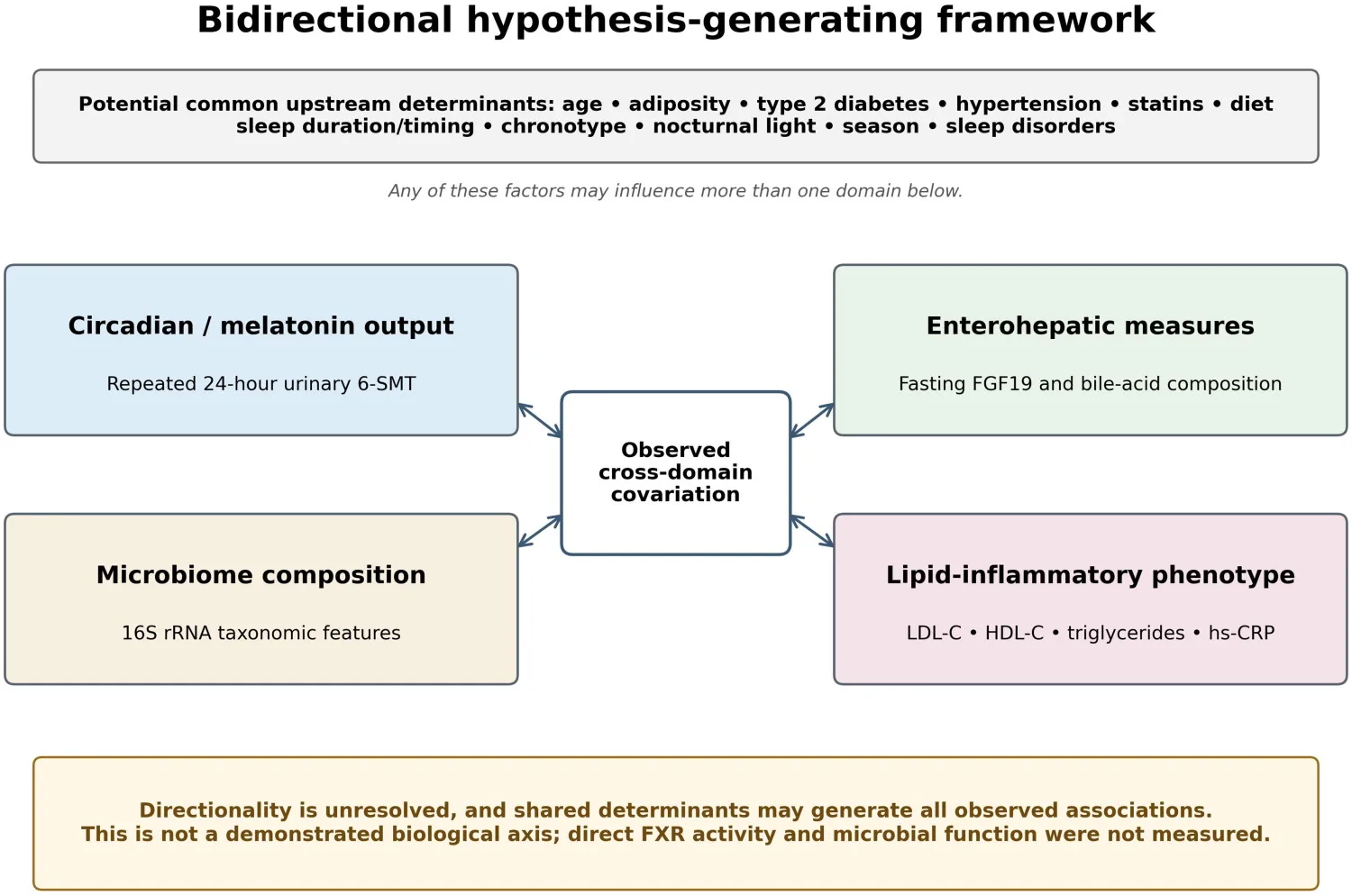
Bidirectional hypothesis-generating framework. Circadian/melatonin output, enterohepatic measures, microbiome taxonomic composition, and the lipid-inflammatory phenotype are shown as interrelated domains with unresolved directionality. Common upstream determinants are displayed explicitly. The diagram is not a demonstrated biological axis; FXR activity and microbial function were not directly measured.

## Discussion

### Principal findings

This study identifies a cross-sectional pattern in adults with dyslipidemia: lower 24-hour urinary 6-SMT was moderately associated with adverse lipid and inflammatory measures; FGF19 was frequently elevated; reported bile-acid composition differed; and selected taxonomic features differed from an unmatched method-matched reference comparator. The statin-stratified mean difference was small and imprecise, and the exploratory model summarized cross-domain covariation without establishing causal independence. The findings may reflect the broader cardiometabolic phenotype represented by obesity, hypertension, diabetes, and medication exposure.

### Lower 6-SMT as an observational cardiometabolic correlate

Melatonin has mechanisms relevant to lipid and vascular biology, including direct and indirect anti­oxidant actions, mitochondrial support, receptor-mediated metabolic effects, and modulation of inflammatory signaling [1–6,12, 38,39]. Lower nocturnal or 24-hour melatonin-metabolite output could therefore be a correlate of altered circadian-redox physiology. The observed correlations were moderate, and this study cannot determine whether lower 6-SMT precedes the cardiometabolic phenotype, results from it, or reflects common determinants such as age, adiposity, diabetes, hypertension, sleep/light exposure, season, or medication.

### Interpretation of FGF19 elevation

In physiological conditions, intestinal FXR activation induces FGF19, which suppresses hepatic bile-acid synthesis and contributes to metabolic regulation [25–28]. In this cohort, elevated circulating FGF19 coexisted with dyslipidemia, altered bile-acid composition, inflammation, and lower 6-SMT. These observations do not reveal whether FGF19 elevation reflects production, clearance, meal-related physiology, compensation, receptor activation, or downstream signaling. Direct FXR activity, receptor occupancy, and signaling efficiency were not measured; mechanistic labels have therefore been removed.

### Microbiome associations and functional inference

The reported differences in C. minuta, F. prausnitzii, and A. muciniphila are biologically contextualized by prior literature [31–34], and experimental work supports possible reciprocal relationships between melatonin biology and the intestinal environment [18,20–24]. However, 16S rRNA profiling primarily provides taxonomic information. Statements about butyrate production, mucosal barrier function, bile-acid conversion, or metabolic activity describe literature-based biological hypotheses, not functions directly measured in this cohort. The targeted taxa were not preregistered or formally prespecified before examination of the microbiome results; they were selected for literature-based metabolic and barrier relevance, and the analysis is not presented as an unbiased whole-microbiome discovery study.

### Meaning and limits of the integrated framework

The conceptual figure deliberately avoids a linear mechanistic sequence. Circadian/melatonin output, enterohepatic measures, microbiome composition, and the lipid-inflammatory phenotype are connected bidirectionally, while common upstream factors are shown as potential shared determinants. This representation matches the cross-sectional evidence: several domains covary, but directionality and coordination as a biological pathway remain unproven.

### Clinical and research implications

These findings should not be interpreted as evidence that melatonin supplementation is established therapy for dyslipidemia. The study also does not establish a clinically meaningful 6-SMT threshold, diagnostic accuracy, test-retest clinical classification, prospective outcome prediction, or incremental value beyond conventional cardiometabolic variables. Repeated 24-hour urinary 6-SMT remains a research measure for prospective validation. Therapeutic implications should be considered only after matched, longitudinal, predictive, and intervention studies and always alongside evidence-based lipid management.

### Strengths

Strengths include prospective consecutive enrollment, a large dyslipidemia cohort, three non-consecutive 24-hour urine collections per participant, a strict creatinine-based collection-validity criterion, immediate unfrozen plasma analysis, complete data for the reported exploratory model, integration of lipid, inflammatory, enterohepatic, and microbiome measures, and method-matched processing of the internal reference comparator. Reporting one focused primary hypothesis, effect estimates with confidence intervals, a statin-stratified comparison, and a transparent post hoc multiplicity sensitivity analysis improves interpretability while preserving the exploratory status of cross-domain analyses.

### Limitations

Limitations are substantial. The analysis is cross-sectional, single-center, and cannot establish causality or temporal ordering. The internal reference cohort was a convenience institutional comparator rather than a concurrently recruited and individually matched control arm. BMI was unavailable, and aggregate sex distribution, comorbidities, medication exposure, smoking, alcohol use, and diet were not available in the verified comparator summary. Residual differences in age, adiposity, diet, lifestyle, and medication may therefore explain part of the reported fold differences. The age-stratified 6-SMT partitions were small and provisional and were not used to define diagnostic accuracy or a universal deficiency category.

The microbiome was assessed by 16S rRNA sequencing rather than metagenomic, metatranscriptomic, or metabolomic profiling, so functional claims are inferential. Habitual sleep duration, chronotype, bedtime, nocturnal light exposure, season, actigraphy, and untreated or undiagnosed sleep disorders were not objectively measured. The dyslipidemia cohort was metabolically heterogeneous: 78% were overweight or obese, 69% had hypertension, 27% had type 2 diabetes, and 73% used statins. The exploratory model did not include every cardiometabolic diagnosis and had no prespecified causal estimand; residual confounding and overadjustment concerns limit coefficient interpretation. FGF19 was measured, but production, clearance, direct FXR activity, and downstream signaling were not. The false-discovery-rate analysis covered the 14 reported comparator tests, not the entire taxonomic output.

### Future directions

Priorities include multicenter replication with a prospectively recruited and individually matched comparator; objective actigraphy, habitual sleep, chronotype, nocturnal light, season, and sleep-disorder assessment; standardized diet and medication characterization; deeper metagenomic, metatranscriptomic, metabolomic, and bile-acid phenotyping; direct FXR-pathway assays; and longitudinal evaluation of whether 6-SMT predicts incident cardiovascular or metabolic outcomes beyond established lipid, inflammatory, and clinical risk markers. Diagnostic-performance and incremental-value analyses should precede therapeutic implications. Any intervention trial should preserve standard evidence-based lipid management.

## Conclusions

In 577 prospectively enrolled adults with dyslipidemia, lower continuous 24-hour urinary 6-SMT was moderately associated with adverse lipid and inflammatory measures and with exploratory FGF19, bile-acid, and taxonomic microbiome features. The unmatched reference comparator, unmeasured circadian factors, broader cardiometabolic phenotype, and cross-sectional design prevent causal, diagnostic, predictive, or therapeutic conclusions. The results define hypotheses for matched and longitudinal validation rather than a demonstrated biological axis.

## Supplementary Material

Supplemental Material

05_Supplementary_Material_29Sep2026.docx

## Data Availability

The data supporting the findings are available from the corresponding author on reasonable request, subject to participant privacy protections, ethics approval, and institutional data governance. Fully identifiable participant-level data are not publicly deposited because of privacy and ethical restrictions.
