# Supplementary material for: Lower 24-hour urinary 6-sulfatoxymelatonin and exploratory bile-acid and microbiome associations in adults with dyslipidemia: a prospectively enrolled cross-sectional study
Source: Ann Med. 2026 Oct 7;58(1):2743367. doi: 10.1080/07853890.2026.2743367 (PMC13647349; doi:10.1080/07853890.2026.2743367)
Supplement: Supplementary file 1 [file IANN_A_2743367_SM8454.docx]

**Lower 24-Hour Urinary 6-Sulfatoxymelatonin and Exploratory Bile-Acid and Microbiome Associations in Adults With Dyslipidemia: A Prospectively Enrolled Cross-Sectional Study**

**Supplementary Material**

*Submission ID: 268706862 | Manuscript ID: IANN-2026-4053 | Further revision: 4 September 2026*

**Scope of this revision.** The supplement now focuses exclusively on the current 577-participant cross-sectional study. The historical chronology and programmatic figures have been removed. All comparator analyses are explicitly labeled exploratory, and no identifiable participant-level data are included.

**All study methods are described in the Materials and methods section of the main manuscript. Supplementary Tables S1 and S2 summarize the eligibility criteria and assay specifications, respectively.**

# Supplementary tables

**Supplementary Table S1. Expanded inclusion and exclusion criteria**

| **Category** | **Criterion** |
| --- | --- |
| Inclusion | Age ≥18 years |
| Inclusion | Documented dyslipidemia by prespecified lipid thresholds or treatment for diagnosed hypercholesterolemia |
| Inclusion | Ability to provide informed consent |
| Inclusion | Willingness to complete repeated 24-hour urine collection |
| Inclusion | Willingness to provide a stool sample |
| Exclusion | Melatonin supplementation within 3 months |
| Exclusion | Melatonin-receptor agonist use |
| Exclusion | Beta-blocker therapy |
| Exclusion | Current antidepressant therapy |
| Exclusion | Sleep disorder under active treatment |
| Exclusion | Recent shift work or trans-meridian travel |
| Exclusion | Antibiotic use within 4 weeks |
| Exclusion | Probiotic supplementation within 4 weeks |
| Exclusion | Known hepatobiliary disease |
| Exclusion | Inflammatory bowel disease |
| Exclusion | Active malignancy or chemotherapy within 12 months |
| Exclusion | Clinically significant renal insufficiency |
| Exclusion | Pregnancy or lactation |
| Exclusion | Known pineal-gland abnormality |
| Clarification | Lipid-lowering treatment was permitted; current statin exposure was recorded and analyzed |

**Supplementary Table S2. Assay specifications and analytical handling**

| **Parameter** | **Method** | **Manufacturer / platform** | **Reference / notes** |
| --- | --- | --- | --- |
| 24-h urinary 6-SMT | Competitive ELISA | IBL International RE54031 | Complete 24-h urine; inter-assay CV 8.2% |
| Serum melatonin | RIA | DIAsource | 03:00 subset n=70; supportive analysis |
| Total cholesterol | Enzymatic colorimetric | Roche Diagnostics | Reference <5.2 mmol/L |
| LDL-C | Direct homogeneous | Roche Diagnostics | Reference <3.0 mmol/L |
| HDL-C | Direct homogeneous | Roche Diagnostics | Reference >1.0 men / >1.2 women mmol/L |
| Triglycerides | Enzymatic colorimetric | Roche Diagnostics | Reference <1.7 mmol/L |
| FGF19 | ELISA | R&D Systems | Stated fasting reference 100-200 pg/mL |
| Total bile acids | Enzymatic cycling | Diazyme | Fasting reference 0-10 μmol/L |
| Bile-acid profile | LC-MS/MS | In-house validated | 15 species quantified |
| 16S rRNA sequencing | Illumina MiSeq V3-V4 | QIIME2 / DADA2 / SILVA v138 | ≥10,000 reads/sample; taxonomic, not functional |
| hs-CRP | Immunoturbidimetric | Roche Diagnostics | Reference <3.0 mg/L |

**Supplementary Table S3. Provisional age-stratified 24-hour urinary 6-SMT reference distribution**

| **Age stratum** | **n** | **Robust center, μg/24 h** | **GSD** | **5th percentile (90% CI)** | **95th percentile (90% CI)** |
| --- | --- | --- | --- | --- | --- |
| 21-44 years | 72 | 20.0 | 1.80 | 7.6 (6.8-8.5) | 52.6 (47.1-59.2) |
| 45-81 years | 48 | 10.5 | 1.95 | 3.5 (3.1-4.1) | 31.5 (28.2-35.8) |

*CLSI EP28-A3c robust method (Horn-Pesce biweight), log transformed; confidence intervals by 10,000 bootstrap resamples. Intervals are provisional and descriptive.*

**Supplementary Table S4. Internal normolipidemic reference-comparator cohort: available and unavailable characteristics**

| **Characteristic** | **Description / data availability** |
| --- | --- |
| Total participants | 120 |
| Age range | 21-81 years |
| Age partitions | 21-44 years: n=72; 45-81 years: n=48 |
| Recruitment source | Institutional volunteers: physicians, faculty members, and students |
| Recruitment period | One year |
| Lipid status | Normolipidemic |
| Renal screening | Normal serum and urinary creatinine |
| Age | Recorded |
| Sex | Recorded; aggregate sex distribution was not available in the verified comparator summary |
| BMI | Not available |
| Comorbidities | Aggregate data not available |
| Medication exposure | Aggregate data not available |
| Smoking and alcohol | Aggregate data not available |
| Dietary characteristics | Aggregate data not available |
| 6-SMT workflow | Same repeated 24-h collection, preanalytical, assay, and laboratory workflow |
| Microbiome workflow | Same personnel, V3-V4 sequencing, QIIME2, DADA2, and SILVA v138 |
| Bile-acid workflow | Same LC-MS/MS method |
| Role in analysis | Internal method-matched reference comparator; not an individually matched control arm |

*Unavailable comparator characteristics were not inferred or imputed. This limitation constrains interpretation of all comparator fold differences.*

**Supplementary Table S5. Descriptive 24-hour urinary 6-SMT values by subgroup and statin sensitivity analysis**

| **Subgroup / contrast** | **n** | **6-SMT, μg/24 h** | **Interpretation** |
| --- | --- | --- | --- |
| 20-29 years | 18 | 12.4 ± 3.8 | Descriptive age subgroup |
| 30-39 years | 62 | 10.8 ± 3.2 | Descriptive age subgroup |
| 40-49 years | 124 | 9.2 ± 2.8 | Descriptive age subgroup |
| 50-59 years | 168 | 7.8 ± 2.4 | Descriptive age subgroup |
| 60-69 years | 142 | 6.4 ± 2.1 | Descriptive age subgroup |
| ≥70 years | 63 | 5.1 ± 1.8 | Descriptive age subgroup |
| Male | 312 | 7.9 ± 2.6 | Descriptive sex subgroup |
| Female | 265 | 8.2 ± 2.8 | Descriptive sex subgroup |
| On statins | 423 | 8.1 ± 2.7 | Statin-stratified sensitivity analysis |
| Statin-naive | 154 | 8.4 ± 2.9 | Statin-stratified sensitivity analysis |
| Difference: statin users - non-users | 577 | -0.30 (95% CI -0.83 to 0.23) | Welch t test; p=0.26 |

*Age and sex rows are descriptive. The statin contrast is post hoc and unadjusted. No universal categorical deficiency classification was applied.*

**Supplementary Table S6. Bile-acid and FGF19 profile**

| **Parameter** | **Internal reference** | **Dyslipidemia** | **Descriptive interpretation** |
| --- | --- | --- | --- |
| Cholic acid (CA) | 0.42 ± 0.18 | 0.68 ± 0.31 | Primary bile acid concentration higher |
| Chenodeoxycholic acid (CDCA) | 0.58 ± 0.24 | 0.89 ± 0.38 | Primary bile acid concentration higher |
| Deoxycholic acid (DCA) | 1.24 ± 0.48 | 0.78 ± 0.34 | Secondary bile acid concentration lower |
| Lithocholic acid (LCA) | 0.18 ± 0.09 | 0.11 ± 0.06 | Secondary bile acid concentration lower |
| Primary/secondary ratio | 1.18 ± 0.32 | 2.84 ± 0.78 | Descriptive compositional ratio; not a direct functional assay |
| Conjugated/unconjugated ratio | 2.42 ± 0.68 | 4.18 ± 1.12 | Descriptive compositional ratio; not a direct BSH assay |
| FGF19, pg/mL | 142 ± 48 | 312 ± 98 | Circulating concentration; mechanism unresolved |

*The reference comparator was unmatched. Ratios are compositional summaries and do not directly measure bacterial conversion, bile salt hydrolase activity, FXR activity, or signaling efficiency.*

**Supplementary Table S7. Alpha-diversity metrics with false-discovery-rate sensitivity analysis**

| **Metric** | **Internal reference (n=120)** | **Dyslipidemia (n=577)** | **Unadjusted p** | **BH q upper bound** |
| --- | --- | --- | --- | --- |
| Shannon index | 3.82 ± 0.41 | 3.24 ± 0.52 | <0.001 | ≤0.0014 |
| Chao1 richness | 412 ± 78 | 328 ± 91 | <0.001 | ≤0.0014 |
| Simpson index | 0.94 ± 0.03 | 0.88 ± 0.05 | <0.001 | ≤0.0014 |

**Supplementary Table S8. Reported species-level microbiome differences with false-discovery-rate sensitivity analysis**

| **Species** | **Internal reference** | **Dyslipidemia** | **Fold difference** | **Unadjusted p** | **BH q / upper bound** |
| --- | --- | --- | --- | --- | --- |
| Christensenella minuta | 0.18 ± 0.09 | 0.02 ± 0.02 | 9.0-fold lower | <0.0001 | ≤0.00047 |
| Faecalibacterium prausnitzii | 8.4 ± 3.2 | 2.1 ± 1.4 | 4.0-fold lower | <0.0001 | ≤0.00047 |
| Akkermansia muciniphila | 2.1 ± 1.2 | 0.3 ± 0.4 | 7.0-fold lower | <0.0001 | ≤0.00047 |
| Bacteroides vulgatus | 4.8 ± 2.1 | 3.2 ± 1.8 | 1.5-fold lower | 0.021 | 0.0245 |
| Ruminococcus bromii | 2.3 ± 1.1 | 1.4 ± 0.9 | 1.6-fold lower | 0.034 | 0.0366 |
| Bilophila wadsworthia | 0.08 ± 0.05 | 0.31 ± 0.18 | 3.9-fold higher | <0.001 | ≤0.0014 |

**Supplementary Table S9. Reported phylum-level microbiome composition with false-discovery-rate sensitivity analysis**

| **Phylum** | **Internal reference** | **Dyslipidemia** | **Unadjusted p** | **BH q / upper bound** |
| --- | --- | --- | --- | --- |
| Firmicutes | 52.3 ± 8.4 | 61.7 ± 9.2 | <0.001 | ≤0.0014 |
| Bacteroidetes | 38.1 ± 7.6 | 28.4 ± 6.8 | <0.001 | ≤0.0014 |
| Actinobacteria | 4.2 ± 2.1 | 5.8 ± 2.4 | 0.042 | 0.0420 |
| Proteobacteria | 3.1 ± 1.8 | 4.9 ± 2.3 | 0.008 | 0.0102 |
| Verrucomicrobia | 2.3 ± 1.4 | 0.8 ± 0.6 | <0.001 | ≤0.0014 |

*Benjamini-Hochberg adjustment was applied across all 14 tests in Tables S7-S9. Boundary substitution for inequality p values yields conservative q-value upper bounds. Statistical retention does not remove confounding from the unmatched comparator.*

**Supplementary Table S10. Exploratory complete-case multivariable model for continuous 24-hour urinary 6-SMT**

| **Predictor** | **Coefficient** | **SE** | **Standardized β** | **P value** | **95% CI** |
| --- | --- | --- | --- | --- | --- |
| Intercept | 18.42 | 1.84 | - | <0.001 | 14.8 to 22.0 |
| Age (per decade) | -0.92 | 0.18 | -0.24 | <0.001 | -1.27 to -0.57 |
| LDL-C (mmol/L) | -1.28 | 0.22 | -0.31 | <0.001 | -1.71 to -0.85 |
| FGF19 (per 100 pg/mL) | -0.84 | 0.16 | -0.22 | <0.001 | -1.15 to -0.53 |
| F. prausnitzii (% abundance) | 0.42 | 0.08 | 0.26 | <0.001 | 0.26 to 0.58 |
| A. muciniphila (% abundance) | 0.68 | 0.19 | 0.18 | <0.001 | 0.31 to 1.05 |
| hs-CRP (mg/L) | -0.38 | 0.11 | -0.14 | 0.001 | -0.60 to -0.16 |
| BMI (kg/m²) | -0.12 | 0.04 | -0.11 | 0.003 | -0.20 to -0.04 |
| Sex (female vs male) | 0.24 | 0.28 | 0.04 | 0.39 | -0.31 to 0.79 |
| Statin use (yes vs no) | -0.18 | 0.31 | -0.02 | 0.56 | -0.79 to 0.43 |

*Model n=577; R²=0.42; adjusted R²=0.40; all variance-inflation factors <2.5; Durbin-Watson statistic 1.92; complete-case analysis; no imputation. This is a cross-domain association model, not a causal or clinical prediction model.*

**Supplementary Table S11. Covariate rationale and limitations of the exploratory model**

| **Component** | **Rationale / interpretive limitation** |
| --- | --- |
| Purpose of model | Cross-domain description of covariation with continuous 6-SMT; not a causal-effect, diagnostic-performance, or incremental-prediction model. |
| Age | Included because 6-SMT varies strongly with age. |
| BMI | Included as a continuous measure of adiposity. |
| Sex | Included as a basic biological covariate. |
| Statin exposure | Included because 73% of the cohort used statins and lipid treatment could affect principal metabolic measures. |
| LDL-C, FGF19, hs-CRP, F. prausnitzii, A. muciniphila | Included to summarize reported cross-domain associations; coefficients are not interpreted as independent causal effects. |
| Hypertension and type 2 diabetes | Characterized descriptively but not added post hoc to the original cross-domain model. No causal estimand or directed acyclic graph was prespecified, and these composite diagnoses overlap with BMI, lipids, inflammation, and medication exposure. Their omission leaves residual confounding and prevents claims of full cardiometabolic adjustment. |
| Model diagnostics | n=577 complete cases; R²=0.42; adjusted R²=0.40; variance-inflation factors <2.5; Durbin-Watson statistic 1.92; no imputation. |
| Interpretive consequence | The table is supplementary and explicitly exploratory. Prospective studies should prespecify a causal framework and sensitivity covariate sets before coefficient stability is interpreted. |

# Supplementary figure legends

Supplementary Figure S1. 24-Hour Urinary 6-SMT by Age Group. Descriptive mean ± SD values by age group. The figure does not define a universal categorical deficiency threshold.

Supplementary Figure S2. Bile-Acid and FGF19 Profile. Dyslipidemia-cohort values expressed as fold differences relative to the unmatched internal reference comparator. Ratios and FGF19 are descriptive; functional mechanism was not directly measured.

Supplementary Figure S3. Alpha-Diversity Metrics. Dyslipidemia-cohort values expressed as percentages of internal-reference values, with raw values shown. For each reported comparison, conservative BH q≤0.0014.

Supplementary Figure S4. Selected Species-Level Relative Abundance. Log-scale comparison of three literature-focused taxa in the internal reference comparator and dyslipidemia cohort. Conservative BH q≤0.00047 for each reported comparison. The comparator was not individually matched.
